# Anti-Colorectal Cancer Activity of Solasonin from *Solanum nigrum* L. via Histone Deacetylases-Mediated p53 Acetylation Pathway

**DOI:** 10.3390/molecules28186649

**Published:** 2023-09-15

**Authors:** Xintian Lan, Meng Lu, Xiaoxue Fang, Yiming Cao, Mingyang Sun, Mengyao Shan, Wenyi Gao, Yuchen Wang, Wenbo Yu, Haoming Luo

**Affiliations:** 1 College of Pharmacy, Changchun University of Chinese Medicine, Changchun 130117, China; lanxintian2022@163.com (X.L.); menglu11161003@163.com (M.L.); fangxiaoxue1996@163.com (X.F.); caoyiming0099@163.com (Y.C.); sunmy0421@163.com (M.S.); shanmengyao213@163.com (M.S.); gaowy@ccucm.edu.cn (W.G.); 2 Key Laboratory of Effective Components of Traditional Chinese Medicine, Changchun University of Chinese Medicine, Changchun 130117, China

**Keywords:** colorectal cancer, *Solanum nigrum* L., Solasonin, HDAC, P53

## Abstract

(1) Background: *Solanum nigrum* L. is a plant of the genus Solanum in the family Solanaceae and is commonly used to treat tumors. Solasonin (SS) is a steroidal alkaloid extracted from *Solanum nigrum* L. that has anti-colorectal cancer (CRC) activity. (2) Methods: Column chromatography, semi-preparative HPLC and cellular activity screening were used to isolate potential anti-CRC active compounds in Solanum nigrum L., and structure identification using ^1^H-NMR and ^13^C-NMR techniques. Expression levels of HDAC in CRC were mined in the UALCAN database. The in vitro effects of SS on SW620 cell line and its mechanism were examined via Western blot, EdU staining, flow cytometry and immunofluorescence. CRC xenograft model and IHC staining were mainly used to evaluate the role of SS in vivo. (3) Results: The results showed that SS was the most potent anti-CRC component in *Solanum nigrum* L., which induced apoptosis and cell cycle arrest in the SW620 cell line. HDAC was highly expressed in CRC. The treatment of SW620 cell line with SS resulted in a significant downregulation of HDAC, an increase in the level of P53 acetylation and a subsequent increase in the level of P21. The in vivo validation results showed that SS could effectively inhibit CRC growth, which was associated with the downregulation of HDAC. (4) Conclusions: SS treatment for CRC mainly works through the induction of apoptosis and cycle arrest, and its mechanism of action is mainly related to HDAC-induced P53 acetylation, and the HDAC/P53 signaling pathway may be a potential pathway for the treatment of CRC.

## 1. Introduction

The incidence of colorectal cancer (CRC), the third most common malignancy and the second most deadly cancer worldwide, is increasing every year [1]. Currently, the treatment of CRC generally includes surgical resection, targeted therapy, radiotherapy and chemotherapy [2]. These approaches effectively inhibit CRC progression, but the development of new therapeutic agents is crucial due to drug resistance and tumor metastasis [3]. Previous studies have shown that numerous natural products (NPs) have different degrees of inhibitory effects on the development of CRC [4,5,6]. Accordingly, it is of great relevance to search for effective components from NPs for the treatment of CRC and to elucidate their mechanisms.

*Solanum nigrum* L., also known as black *Solanum nigrum* L., is an annual herb of the genus Solanum in the family Solanaceae [7], which was first recorded in the Compendium of Materia Medica [8]. The whole herb of *Solanum nigrum* L. has good efficacy in dissipating stasis and eliminating swelling, clearing heat and removing toxins, and is commonly used in the treatment of aphthous ulcers, eczema of the skin, urinary tract infections, bacillary dysentery, prostatitis, chronic bronchitis, etc. [9]. Modern studies have found that *Solanum nigrum* L. contains alkaloids, steroidal saponins, coumarins, polysaccharides, a variety of vitamins and amino acids. Pharmacodynamic studies have shown that *Solanum nigrum* L. has a variety of biological activities such as antitumor, immunomodulation, antibacterial and antiviral [10]. The chemical composition of *Solanum nigrum* L. consists of steroids, organic acids, lignans and other compounds [11]. Solasonin (SS) is a steroidal alkaloid obtained from *Solanum nigrum* L. [12] and can inhibit the activity of many tumors, such as gastric cancer [13], lung cancer [14], liver cancer [15] and CRC [16]. However, there are fewer studies on the inhibition of CRC activity by SS, and the mechanism of action still needs to be further elucidated.

Histone deacetylases (HDAC) are marker enzymes for post-translational modifications of histones that can promote CRC development by participating in epigenetic pathways that mediate the inactivation of multiple tumor suppressor genes [17,18]. The HDAC family has 18 isoforms, which can be divided into four classes, class I HDACs (HDAC1, HDAC2, HDAC3 and HDAC8) are involved in the regulation of cell cycle progression, DNA repair and apoptosis [19]. p53 is a typical tumor suppressor downstream of HDAC, and acetylated p53 can trigger death receptor-induced apoptotic pathways [20,21]. In addition, the cell cycle protein-dependent kinase (CDK) inhibitor p21 is a downstream target gene of p53 and can block the transition from G1 to S phase of the cell cycle [22]. A growing number of studies have found that the HDAC-regulated p53 pathway plays an important role in CRC. For example, HDAC1 and 2 antagonize the tumor suppressor p53 to regulate p21, which in turn induces CRC cell cycle arrest and apoptosis [23]. The inhibition of HDAC improves epigenetic regulation in CRC cells with p53 mutations [24].

In this study, we aimed to investigate the anticancer effects of SS on CRC in vitro and in vivo and to elucidate the potential mechanisms. First, we obtained the activity-oriented isolation of seven potential anti-CRC active compounds in *Solanum nigrum* L., among which, SS inhibited CRC activity the best. An analysis of the UALCAN database showed that HDAC was highly expressed in CRC. Subsequently, it was confirmed via cell phenotyping experiments that SS could inhibit SW620 cell growth and induce apoptosis by regulating cell cycle protein-dependent kinase 2 (CDK2) and p21. Moreover, SS inhibited HDAC expression, thus exerting significant anti-CRC activity. This study comprehensively demonstrated the potential value of SS in CRC treatment and indirectly demonstrated that SS is an HDAC inhibitor for the treatment of CRC.

## 2. Results

### 2.1. Anti-CRC Activity-Guided Isolation and Purification of Solanum nigrum L.

**Figure 1:**
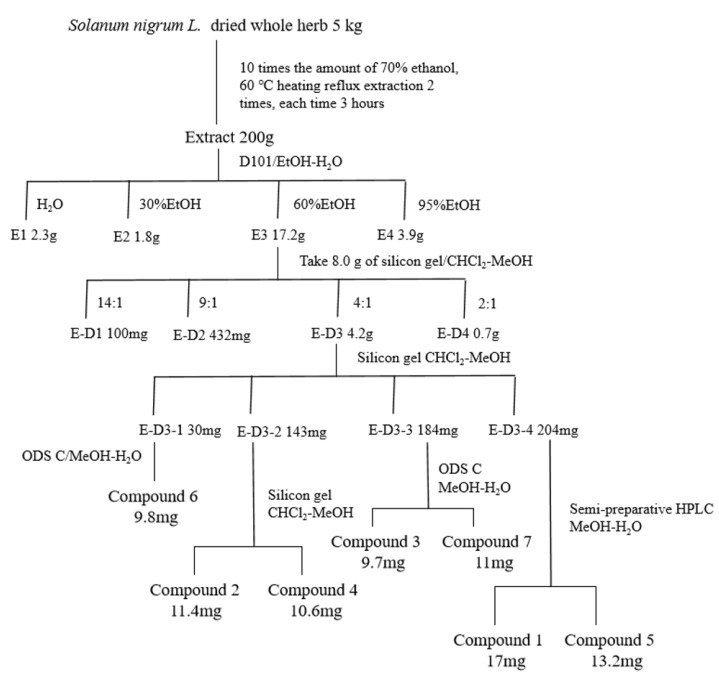
Isolation and purification of *Solanum nigrum* L. guided by anti-CRC activity.

**Figure 2:**
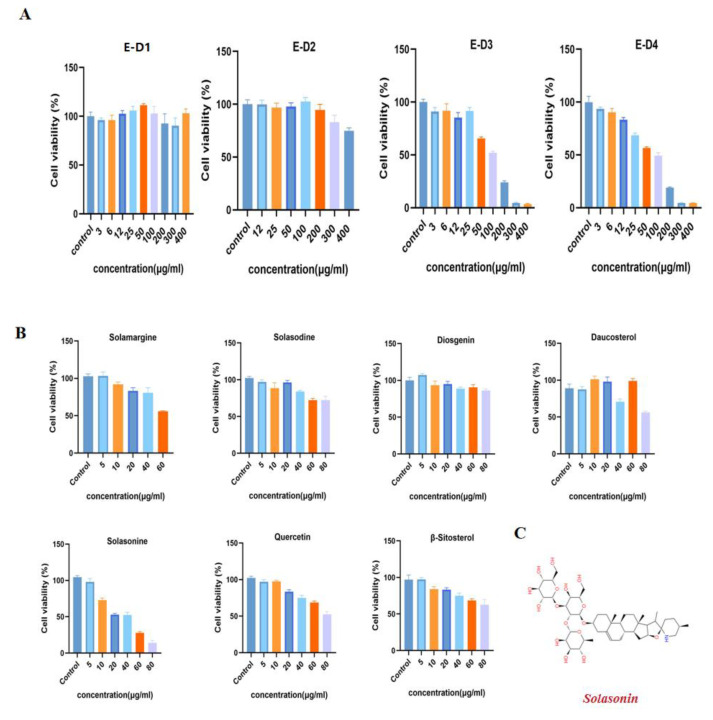
Isolation and purification of *Solanum nigrum* L. guided by anti-CRC activity. (**A**) Effect of components E-D1~E-D4 on SW620 cell viability. The cells were treated with gradient concentrations of E-D1~E-D4 for 24 h and cell viability was measured by MTT. (**B**) Effect of compounds **1**~**7** on SW620 cell viability. (**C**) Chemical structure of SS. All experiments were performed three times independently.

We performed preliminary extraction and isolation of *Solanum nigrum* L. to obtain four fractions E-D1-D4 (Figure 1). The in vitro anti-CRC activity screening revealed that fractions E-D1 and E-D2 had no effect on tumor activity, while fractions E-D3 and E-D4 both had inhibitory ability on tumor activity with IC50 values of 102 µg/mL and 100 µg/mL, respectively (Figure 2A). We then isolated and purified E-D3, which had better inhibitory activity, and obtained seven monomeric compounds. The ^1^H-NMR and ^13^C-NMR spectra identified Solamargine, Solasodine, Diosgenin, Daucosterol, Solasonine, Quercetin and Sitosterol (Figure S1). MTT activity screening revealed that all compounds except Diosgenin had CRC inhibitory ability, with SS (Figure 2C) showing the strongest inhibitory ability against CRC (Figure 2B).

### 2.2. SS Inhibited the Proliferation of CRC Cells

**Figure 3:**
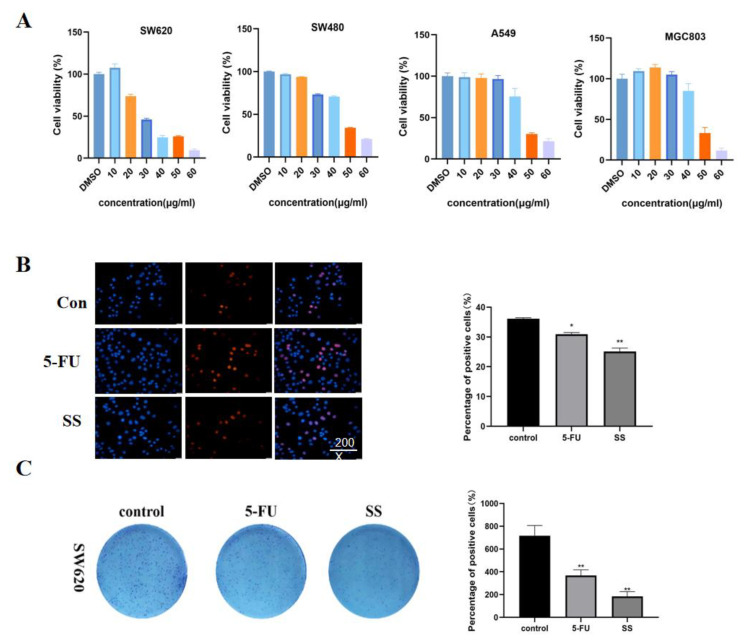
SS inhibited the proliferation of CRC cells. (**A**) Cell viability of SW620, SW480, A549 and MGC803 cells after SS treatment. The cells were treated with different concentrations of SS and equal amounts of DMSO for 24 h. Cell viability was detected using CCK8. (**B**) EdU staining results showed that SS significantly inhibited the proliferation of SW620 cells. Drugs were added as per SS (40 μM) and 5-FU groups (0.7 mM), incubated for 48 h and assayed using the EdU kit. (**C**) Images of colony formation showed that SS treatment significantly reduced the number of colonies. Plate clone formation experiments were performed by adding the drug in the blank group, SS group (40 μM), and 5-FU group (0.7 mM). All experiments were performed three times independently. * *p* < 0.05, ** *p* < 0.01.

First, the cytotoxicity of SS on CRC cells SW620, SW480, A549 and MGC803 was detected using the CCK-8 method. Different concentrations of SS (10, 20, 30, 40, 50 and 60 μM) were given to the four CRC cells for 24 h. The cell survival rate was compared with the control group. The results (Figure 3A) show that SS significantly inhibited the proliferation of CRC cells in a concentration-dependent manner, and IC50 values of SS on SW620, SW480, A549 and MGC803 cells were 35.52 μM, 44.16 μM, 44.18 μM and 46.72 μM, respectively. We found that the inhibitory effect of SS on SW620 was the most sensitive, so we selected it for the follow-up experiments. Based on the results of EdU staining (Figure 3B), it is clear that SS significantly inhibited the proliferation of SW620 cells, and 5-FU was the positive control. In addition, SS treatment significantly reduced the number of colonies compared with the control (Figure 3C). The above results confirmed that SS inhibited the proliferation of SW620 cells in vitro.

### 2.3. SS Induces Cell Cycle Arrest and Apoptosis in CRC Cells

**Figure 4:**
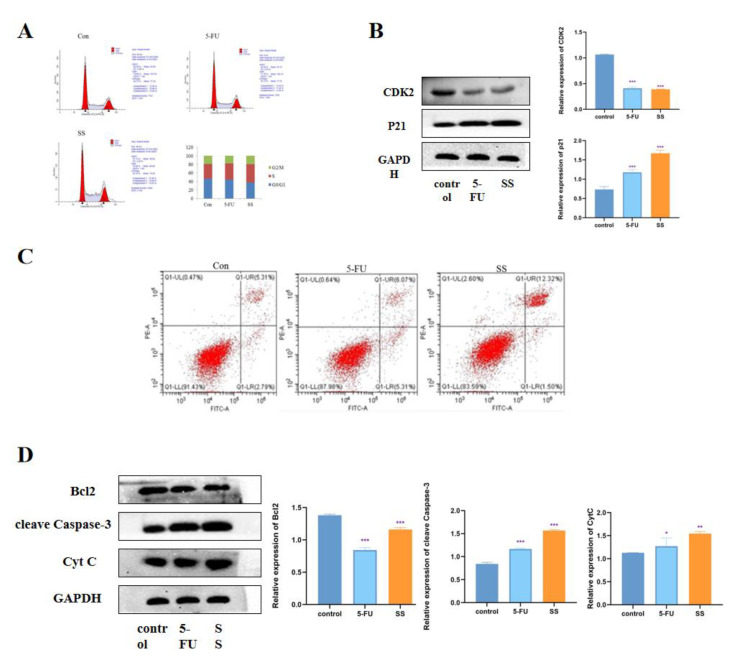
SS induces cell cycle arrest and apoptosis in CRC cells. (**A**) Flow cytometry analysis to determine the cycle of SW620 cells after 4 h treatment in blank group, SS group (40 μM) and 5-FU group (0.7 mM). (**B**) Western blot analysis of CDK2 and p21 expression levels in SW620 cells after 24 h of incubation in the blank group, SS group (40 μM) and 5-FU group (0.7 mM). (**C**) Flow cytometry analysis to determine the apoptosis of SW620 cells in the blank group, SS group (40 μM) and 5-FU group (0.7 mM) after 24 h of culture. (**D**) Western blot analysis of the expression levels of cleave Caspase-3, Bcl2 and Cyt C in SW620 cells after 24 h of culture in the blank group, SS group (40 μM) and 5-FU group (0.7 mM). All experiments were performed three times independently. * *p* < 0.05, ** *p* < 0.01, *** *p* < 0.001.

To further investigate the mechanism by which SS inhibits the proliferation of CRC cells, we examined the effect of SS on the cell cycle. The results are shown in Figure 4A. In SW620 cells, the percentage of S-phase cells increased significantly after SS treatment, while the percentage of G0/1 phase and G2/M phase cells decreased significantly. This indicates that SS blocked the cell cycle in S phase, thereby inhibiting the proliferation of SW620 cells. Western blot results showed (Figure 4B) that the expression of CDK2 was significantly decreased after SS treatment, while the expression level of p21 was significantly increased, with statistically significant differences (*p* < 0.05). This further confirmed that SS inhibited CRC cell proliferation by blocking the cell cycle in S phase. To assess the effect of SS on apoptosis, flow cytometry analysis was performed using Annexin V-FITC/PI double staining. As shown in Figure 4C, after 24 h of SS treatment, apoptosis was significantly increased in the treated group compared with the blank group cells. Then, Western blot was used to detect the expression of cleave Caspase-3, Bcl2 and cyt C. From the results in Figure 4D, it can be seen that SS treatment significantly increased the expression of cleave Capase-3 and decreased the protein expression of Bcl2 compared with control cells, but SS treatment had no effect on the expression of Cyt C. The above results suggest that SS may induce apoptosis in SW620 cells through the P53 pathway.

### 2.4. Class I HDAC Is Highly Expressed in CRC Cells

**Figure 5:**
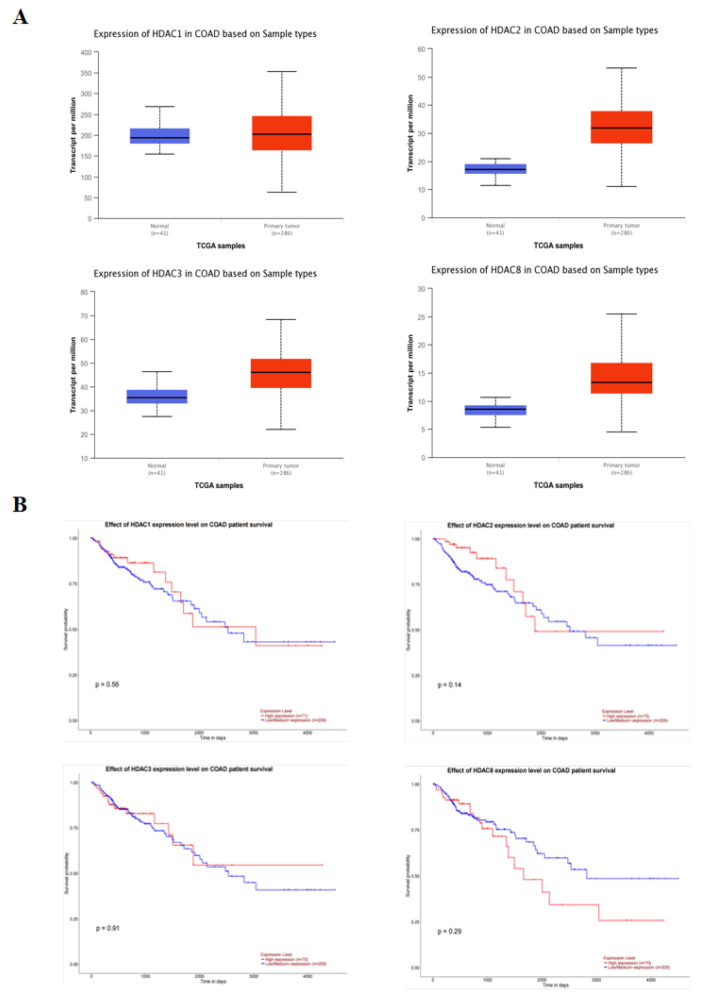
Class I HDAC is highly expressed in CRC cells. (**A**) Expression of class I HDAC in CRC patients in the UALCAN database. (**B**) Impact of class I HDAC in CRC patients in the UALCAN database on survival.

The relationship between the expression of class I HDAC and the probability of survival in 286 colonic adenocarcinoma (COAD) patients was analyzed using the UALCAN database. The results of Figure 5A show that the expression of class I HDAC was significantly increased in cancer patients. Moreover, there was no significant change in survival probability between the low and high expression groups, but a decreasing trend in survival probability could be seen in the high HDAC expression group (Figure 5B).

### 2.5. Downregulation of Class I HDAC Is Involved in SS-Mediated Anti-CRC Effects

**Figure 6:**
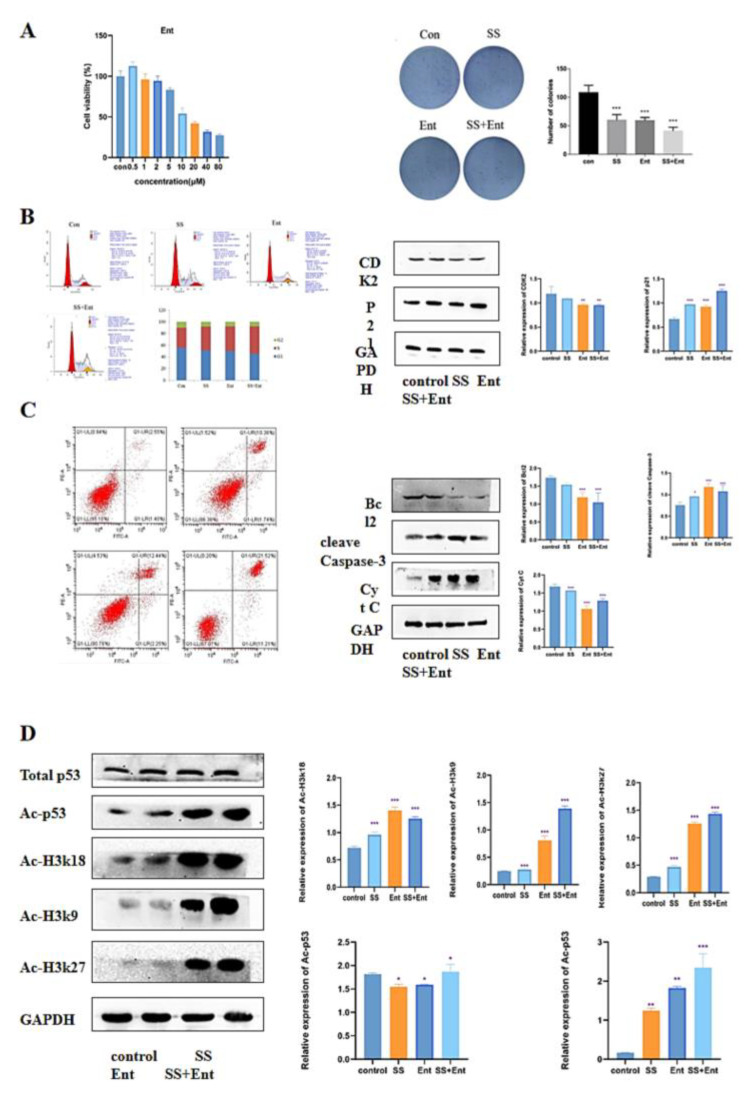
SS-mediated anti-CRC effect acts mainly through class I HDAC-induced P53 acetylation. (**A**) Images of the effect of different concentrations of Ent on the viability of SW620 cells and colony formation. (**B**) Flow cytometry analysis to determine the cycle of SW620 cells after treatment for 24 h. Western blot analysis of the expression levels of CDK2 and p21 in SW620 cells after 24 h of post-administration culture. (**C**) Flow cytometry analysis to determine the apoptosis of SW620 cells after 24 h of post-administration culture. Western blot analysis of the expression levels of cleave Caspase-3, Bcl2 and Cyt C in SW620 cells after 24 h of post-administration. (**D**) Western blot analysis of histone H3K27, H3K18, H3K9 and H4 acetylation as well as the expression levels of p53 acetylation and total p53 content in SW620 cells. The above experiments were grouped according to blank group, SS group (40 μM), Ent group (15 μM), SS (40 μM) + Ent (15 μM) group, and all experiments were performed three times independently. * *p* < 0.05, ** *p* < 0.01, *** *p* < 0.001.

To confirm whether HDAC is a potential target for SS in CRC treatment, we conducted follow-up experiments. We first examined the effect of Ent, a class I HDAC inhibitor, on SW620 activity using the CCK-8 assay, and it was shown in Figure 5A that Ent inhibited SW620 cell activity in a dose-dependent manner with an IC50 value of 17 µM. Next, a plate clone formation assay was used to examine the proliferation of SW620 after 24 h of treatment with SS. The results showed that SS decreased the number of colonies and colony size of SW620 cells; the co-administration of SS with Ent significantly inhibited the number of cell colonies and colony size compared with SS alone, and these results indicated that SS and Ent had a synergistic inhibitory effect on tumor cell proliferation (Figure 6A). Meanwhile, in SW620 cells, the percentage of S-phase cells significantly increased after SS treatment, while the percentage of G0/1 and G2/M-phase cells significantly decreased (Figure 6B). Western blot results showed (Figure 6B) that the expression of CDK2 significantly decreased after SS treatment, while the expression level of p21 significantly increased, with statistically significant differences (*p* < 0.05). This indicates that SS and Ent synergistically blocked the cell cycle in S phase, thereby inhibiting SW620 cell proliferation. In addition, flow cytometry results showed (Figure 6C) that apoptosis was significantly increased in the treated group compared with the blank group cells. Western blot results showed (Figure 6C) that both SS and Ent downregulated Bcl-2 protein expression levels and upregulated Caspase-3 and Cyt C protein expression levels in tumor cells, and SS and Ent had a synergistic effect in inducing apoptosis.

### 2.6. SS Acts through Class I HDAC-Induced P53 Acetylation

In the previous results, class I HDAC had a significant role in SS treatment. The apoptosis-related target gene p53 is an important target of HDAC. Therefore, we hypothesized that the effect of SS-induced apoptosis in CRC cells was associated with acetylation of p53. Western blot confirmed that SS decreased the expression level of class I HDAC proteins and increased acetylation of histones H3K27, H3K18, H3K9 and H4 in SW620 cells. Next, we evaluated the effect of SS on p53 acetylation in SW620 cells. The results showed that SS increased the acetylation of p53 compared to the control, but barely affected the expression of p53 in SW620 cells, which was associated with reduced levels of HDAC (Figure 6D).

### 2.7. SS Inhibited the Growth of SW620 Xenograft Mouse Model

**Figure 7:**
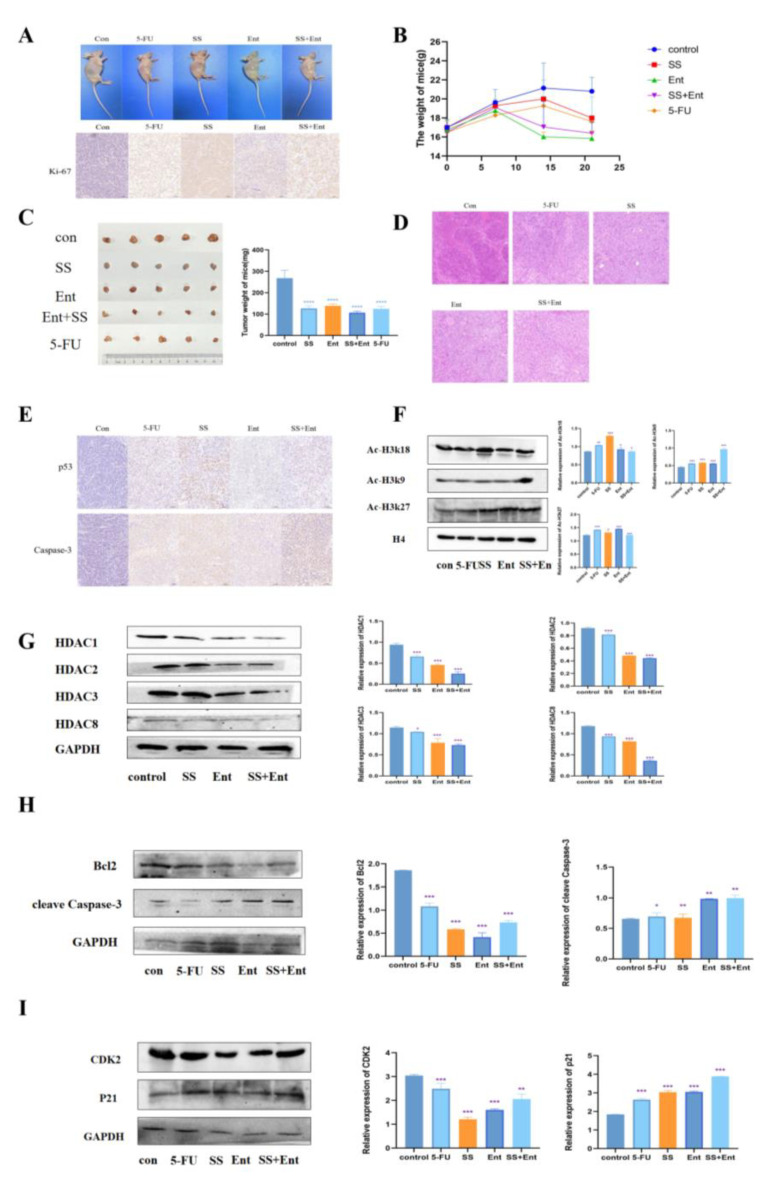
SS acts through class I HDAC-induced p53 acetylation. (**A**) IHC assessment of the effect of SS on Ki-67 expression levels in CRC cell nude mice subcutaneously transplanted with tumors. (**B**) Weight–time curves were plotted based on the body weight of nude mice measured during SS administration. (**C**) Tumor appearance and mean weight of tumors in each drug group. (**D**) HE staining to detect the inhibitory effect of SS on subcutaneously transplanted tumors in CRC cell nude mice. (**E**) IHC detection of SS-induced apoptosis of tumor cells. (**F**) Western blot analysis of the effects of acetylation of H3K27, H3K18, H3K9 and H4 in CRC cell nude mice subcutaneously transplanted tumors in the blank group, 5-FU group (25 mg/kg), SS group (50 mg/kg), Ent group (25 mg/kg) and SS (50 mg/kg) + Ent (25 mg/kg) group. (**G**) Protein blot analysis to detect the protein expression of class I HDAC in SS, Ent and SS + Ent-treated CRC cells. (**H**) Western blot analysis of the expression levels of cleave Caspase-3 and Bcl2 in SW620 cells 24 h after drug administration. (**I**) Western blot analysis of CDK2 and p21 expression levels in SW620 cells 24 h after drug administration in culture. All experiments were performed three times independently. * *p* < 0.05, ** *p* < 0.01, *** *p* < 0.001, **** *p* < 0.0001.

To confirm the role of SS in vivo, we established a human CRC xenograft mouse model (Figure 7A). According to the body weight and tumor weight of nude mice measured during SS administration, SS treatment reduced CRC tumor growth (Figure 7B,C). The IHC staining results showed that the expression of Ki-67 was significantly reduced in each treatment group (Figure 7A), indicating that SS inhibited tumor growth in vivo by suppressing tumor cell proliferation. The HE staining results showed (Figure 7D) that the tumor cells in the control group grew in a good state in vivo, with the number of cells being high, closely arranged, with large cell size, round nuclei, two to three nucleoli visible and in pathological nuclear division phase. After the administration of SS, the number of cells was significantly reduced, and some cells were seen to be reduced in size, with a deepened cytoplasmic staining, smaller nuclei, concentrated chromatin and unclear nucleoli, and some cells showed cytoplasmic sparing, indicating apoptosis. In addition, SS was able to significantly upregulate apoptosis protein Caspase3 and p53 protein expression (Figure 7E). Then, we examined the protein levels of intratumoral class I HDAC and found that SS could inhibit the levels of intratumoral class I HDAC, which in turn upregulated the acetylation levels of histone H3k9, H3k18 and H3k27, which was consistent with the in vitro results (Figure 7F,G). Finally, we examined in vivo the expression of apoptosis and cycle-related proteins, and the results were consistent with that in vitro (Figure 7H,I). In summary, these results suggest that the SS-induced downregulation of HDAC is associated with its anti-CRC effect in vivo.

### 2.8. SS Evaluation of Docking with Class I HDACs

**Figure 8:**
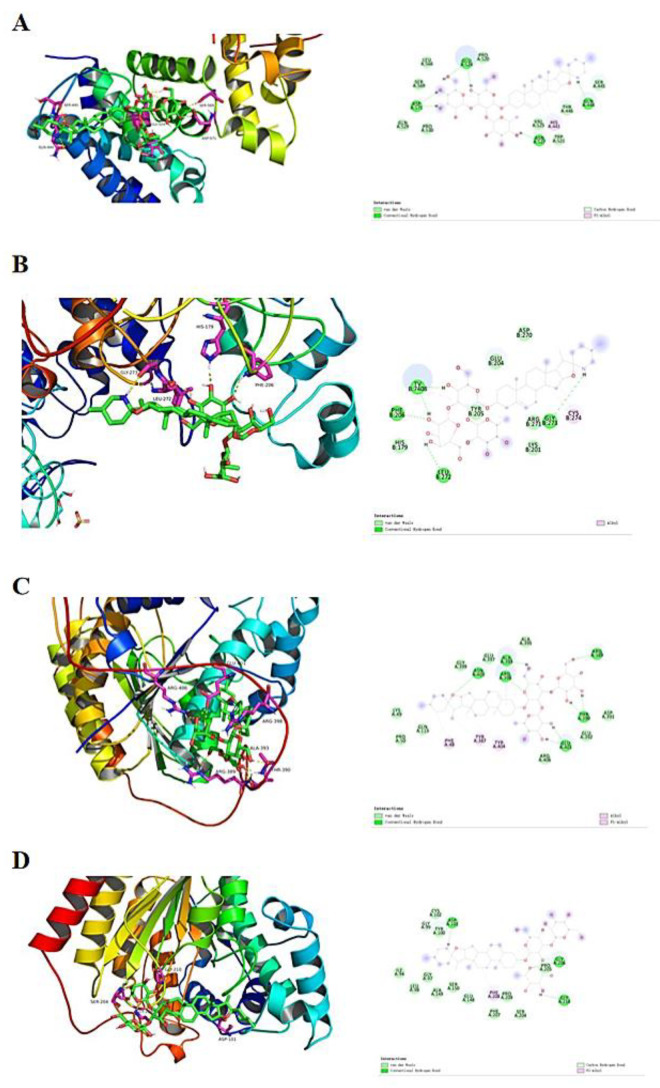
Molecular docking results. (**A**) SS docked conformation with HDAC1. (**B**) SS docked conformation with HDAC2. (**C**) SS docked conformation with HDAC3. (**D**) SS docked conformation with HDAC8.

Experiments were performed with SS to dock HDAC1, HDAC2, HDAC3 and HDAC8 proteins, respectively. As shown in Table 1, SS and all three target proteins were spatially matched to each other and the docking conformation was stable (Figure 8). The compounds exhibited strong binding energy by forming hydrogen bonding forces with key amino acids that are neutral in activity. For the HDAC1 receptor, SS formed five hydrogen bonding forces with GLU524, ASP571, ASN527, HIS441 and GLN444, respectively (Figure 8A). For HDAC2 receptor, SS formed six hydrogen bonding forces with TV, PHE, LEU, TYP, CYS and GLY, respectively (Figure 8B). For HDAC3 receptor, SS formed nine hydrogen bonding forces with ALA393, ARG398, ARG389, ASN403, PHE48, TYR387, TYR404, THR390 and GLU401, respectively (Figure 8C). For the HDAC8 receptor, SS formed five hydrogen bonding forces with ASP101, GLY99, PHE208, GLY210 and GLY206, respectively (Figure 8D). In summary, SS formed hydrogen bonding forces with HDAC receptors and exhibited the binding ability of class I HDAC targets, and thus had an anti-CRC effect.

## 3. Discussion

CRC is a gastrointestinal malignancy caused by various genetic and epigenetic changes in the intestinal epithelium of the colon and rectum, the incidence of which is increasing year by year [25]. Previous studies have found that *Solanum nigrum* L. is widely used clinically for anti-tumor purposes, such as pancreatic cancer [26], gastric cancer [27], glioma [28] and CRC [29], among others. Based on the screening of anti-CRC active components in *Solanum nigrum* L., we found that SS has good anti-CRC activity. However, the anti-CRC activity of SS has been less studied and there are almost no studies for epigenetic aspects. It is well known that epigenetic plays an important role in tumor development. Thus, the present study aimed to investigate the epigenetic pathways through which SS exerts its anti-CRC effect.

Next, through an in vitro flow cytometry analysis of SS-treated cells, we identified the cell cycle as a possible target for SS to inhibit CRC cell proliferation. As expected, SS induced S-phase block in CRC cells. In addition, we measured the effect of SS on cell cycle-related proteins. Our results showed that SS downregulated CDK2 and upregulated p21. Flow cytometry results showed that SS could induce apoptosis in CRC cells. Moreover, SS also inhibited Bcl-2 and upregulated the expression levels of caspase3 and Cyt C. In vivo, we established a human CRC xenograft mouse model, and SS reduced CRC tumor growth and inhibited tumor cell proliferation. HE staining results also indicated that apoptosis occurred after SS treatment. In addition, SS significantly upregulated the expression of apoptotic proteins cleave Caspase3 and p53 protein. Thus, it is clear that SS can inhibit CRC value-added and induce apoptosis and cycle arrest both in vivo and ex vivo. Based on this, we hope to find a key fulcrum that can both regulate apoptosis and induce cycle arrest, which is the question of interest to us. We identified p53 as this key target, which can both regulate apoptosis and induce cycle arrest. Next, we investigated the upstream and downstream proteins of p53. Surprisingly, the epigenetic enzyme HDAC can regulate p53 acetylation to participate in processes such as in the regulation of cell cycle progression and apoptosis [30,31]. When HDAC is inhibited, p53 induces apoptosis through trans-activation, which is subsequently accompanied by p21-induced cell cycle arrest [24,32]. We speculate that the HDAC/P53 signaling pathway may be a potential pathway for the treatment of CRC.

To confirm this conjecture, we performed a follow-up experiment. Analysis of the relationship between class I HDAC expression and survival probability in 286 COAD patients from the UALCAN database showed that class I HDAC expression was significantly increased in CRC patients. In vitro studies confirmed that SS can synergize with Ent to exert anti-CRC effects. Western blot confirmed that SS decreased class I HDAC protein expression levels and increased p53 acetylation in vivo, but barely affected p53 expression in SW620 cells. Finally, in vivo, we examined the expression of apoptosis and cycle-related proteins, and the results were consistent with those in vitro.

In conclusion, the up-expression of HDACs in malignant cancer cells leads to an imbalance in cell proliferation, differentiation and inactivation of tumor suppressors [33]. Thus, HDACs have been used as anticancer targets and are involved in different cancer types such as lymphoma, non-small cell lung cancer, colorectal cancer and prostate cancer [34,35,36]. *Solanum nigrum* L. is rich in resources, with simple processing, is easy to obtain and apply, has a wide source and a remarkable therapeutic effect as well as only few toxic side effects [11]. Based on these advantages, it is our goal to continue to study the anticancer efficacy of SS, especially in various solid tumors, in combination with anticancer drugs with different mechanisms of action, as well as to determine the constitutive relationship for new drug design. SS as an HDAC inhibitor can effectively inhibit over-activated HDAC, leading to tumor cell blockade, the enhancement of apoptosis and the promotion of cell cycle arrest, which are expected to provide a novel vision for the construction of next-generation HDAC inhibitors.

## 4. Materials and Methods

### 4.1. Reagents

The EdU kit (Cat No: C10310-1) was purchased from RiboBio, Guangzhou, China, and the extreme ultrasensitive ECL chemiluminescence kit (Cat No: P0018FM) was purchased from Beyotime, China. Trypsin (Cat No: T1300), MTT reagent (Cat No: M8180), cell-grade dimethyl sulfoxide DMSO (Cat No: D8371) and Rainbow 180 Broad Spectrum Protein Marker (Cat No: PR1910) were purchased from Solarbio, Beijing, China. DMEM high glucose medium (Cat No: SH30243.01) was purchased from Gibco, New York, NY, USA, and 100 U penicillin–streptomycin solution (Cat No: SV30010) was purchased from Hyclone, Los Angeles, CA, USA. PVDF membranes, 5-fluorouracil (5-FU) and Entinostat (Ent) were obtained from MERCK, Darmstadt, Germany. Bcl2 antibody (Cat No: 68103-1-Ig), Caspase-3 antibody (Cat No: 66470-2-Ig), Cytochrome C (Cyt C) antibody (Cat No: 10993-1-AP), p53 antibody (Cat No: 60283-2-Ig), p21 antibody (Cat No: 10355-1-AP), CDK2 antibody (Cat No: 10122-1-AP), HDAC1 antibody (Cat No: 66085-1-Ig), HDAC2 antibody (Cat No: 67165-1-Ig), HDAC3 antibody (Cat No: 67151-1-Ig) and HDAC8 antibody (Cat No: 17548-1 AP) were purchased from Proteintech, Wuhan, China. H4 antibody (Cat No: PTM-1009), H3K18 antibody (Cat No: PTM-114), H3K27 antibody (Cat No: PTM-5010) and H3K9 antibody (Cat No: PTM-7094) were purchased from Jingjie PTM BioLab, Hangzhou, China.

### 4.2. Collection of Plants and Preparation of Extracts

*Solanum nigrum* L. (plant name has been checked by http://www.theplantlist.org, accessed on 3 September 2022), purchased from Jilin North Herb Company, was identified by Professor Lili Weng of Changchun University of Traditional Chinese Medicine as *Solanum nigrum* L. of the genus Solanaceae. *Solanum nigrum* L. (production lot no.: 20200712) was kept in the Laboratory of Traditional Chinese Medicine Chemistry, College of Pharmacy, Changchun University of Chinese Medicine. The filtrate was concentrated under reduced pressure and the crude extract was obtained as an infusion. The infusion was dispersed with water and extracted with equal amounts of petroleum ether to obtain aqueous and petroleum ether extracts. The aqueous extract was placed in a D101 macroporous resin column (Tianjin Guangfu Fine Chemical Research Institute, Tianjing, China) and eluted with ethanol/water (0:100, 30:70, 60:40, 95:5) to obtain four fractions E1~E4. Four fractions E-D1~E-D4 were obtained by taking 8.0 g of fraction E3, placing it in a silica gel column and eluting it with dichloromethane/methanol (Energy Chemical, Jiaxing, China) (14:1, 9:1, 6:1, 2:1). Seven compounds were separated via silica gel column chromatography, ODS column chromatography and reversed-phase HPLC. The detailed separation process is shown in Figure 1 Compound **1** obtained from the separation and purification was dissolved with deuterated methanol (Macklin, Guangzhou, China), and compounds **2**–**7** were dissolved with deuterated pyridine (Macklin, Guangzhou, China) and analyzed using ^1^H-NMR and ^13^C-NMR.

### 4.3. Cell Line

SW620, SW480, A549 and MGC803 cells were provided by Procell, Wuhan, China. The culture media used for the experiments all contained 10% fetal bovine serum, 1% penicillin–streptomycin (Hyclon, Logan, UT, USA) and 89% DMEM medium (Hyclon, Logan, UT, USA). The incubator conditions were 5% CO_2_, 95% air and 37 °C.

### 4.4. Animals

A total of 35 6-week-old specific pathogen-free (SPF) male BALB/c nude mice were purchased from Speffer, China. The mice were randomly divided into 5 cages and given sufficient feed and drinking water. The general status of the nude mice was observed and recorded every other day, and the body weights of the mice were weighed and recorded. After the nude mice had adapted to the environment for one week, a subcutaneous transplantation tumor model was prepared. All animal experimental procedures and processes involved in this experiment were performed in accordance with the requirements of the Ethics Committee of Changchun University of Traditional Chinese Medicine, ethics number: 2021276.

### 4.5. MTT Assay

The effects of E-D1~E-D4 and compounds **1**–**7** on the cell viability of SW620 were assayed using MTT powder. Cells were inoculated at 8 × 10^3^ cells/well in 96-well plates overnight and treated with drugs for 24 h. Then, cells were incubated with MTT reagent at 37 °C for 4 h. The supernatant was gently discarded and DMSO was added for lysis. Cell viability was calculated by measuring the absorbance at 490 nm using an enzyme marker (Molecular Devices, Shanghai, China).

### 4.6. Cell Count Kit-8 (CCK-8) Assay

The effect of SS on the viability of SW620, SW480, A549 and MGC803 cells was assayed using CCK-8 (Invigentech, Shanghai, China). Cells (8 × 10^3^) were inoculated in 96-well plates overnight and treated with drugs for 24 h. Then, cells were incubated with CCK-8 for 1 h at 37 °C. Cell viability was calculated by measuring absorbance at 450 nm with an enzyme marker (Molecular Devices, Ismaning, Germany).

### 4.7. EdU Assay

In the EdU assay, SW620 cells were inoculated at 8 × 10^3^ cells/well in a 96-well plate and cultured overnight. Drugs were added according to the macadamia group (40 µM) and 5-FU group (0.7 mM), and the incubation was continued for 48 h. Cell viability was detected using the EdU assay kit. The staining was examined under a microscope (Keyence, Osaka, Japan).

### 4.8. Colony Formation Assay

SW620 cells were inoculated overnight in 6-well plates at 2.5 × 10^5^ cells/well, and the culture was continued for 24 h after the addition of drugs, and then replaced with conventional medium for 15 days. Next, the 6-well plates were washed with PBS and the cells were stained with 0.1% crystal violet for 30 min at room temperature. After washing three times with PBS, the cells were air-dried and the experimental data were analyzed using camera photography and ImageJ.

### 4.9. Cell Cycle Assay

SW620 cells were inoculated at 2.5 × 10^5^ cells/well in a 6-well plate overnight, drug was added, and culture was continued for 24 h. Cells were collected and washed twice with pre-chilled PBS. Then, the cells were assayed for DNA content via flow cytometry, and the distribution of G0/G1 phase, S phase or G2/M phase cells was analyzed using Flow Jo 7.6.2 software.

### 4.10. Apoptosis Detection Assay

The Annexin V-FITC/PI Apoptosis Detection Kit (Beyotime, Shanghai, China) was used to analyze the apoptosis of SW620 cells after SS treatment. Cells (2.5 × 10^5^) were inoculated in 6-well plates overnight, drugs were added, and culture was continued for 24 h. Cells were collected and washed twice with pre-chilled PBS. The cells were then treated according to the instructions of Annexin V-FITC/PI Apoptosis Detection Kit, and apoptosis was detected using flow cytometry (Beckman, Shanghai, China).

### 4.11. Network Target Verification

Box plots of class I HDAC expression and KM survival curves were plotted by accessing publicly available cancer histology data (TCGA, MET500 CPTAC and CBTTC) using the UALCAN database (http://ualcan.path.uab.edu/index.html, accessed on 23 March 2023). Searches were performed using colon adenocarcinoma (COAD) as a keyword to obtain class I HDAC expression, survival time and survival status in 41 normal subjects and 286 cancer patients.

### 4.12. Western Blot Assay

SW620 cells were inoculated at 2.5 × 10^5^ cells/well in a 6-well plate overnight, drugs were added, and the culture was continued for 24 h. The cells were collected, washed twice with pre-chilled PBS, added 2 × loading for mechanical sonication, and assayed for protein content using immunoblotting. Firstly, SD polyacrylamide gel was passed for separation, and when bromophenol blue ran to the bottom of the gel, it was transferred to PVDF membrane, and PVDF membrane was closed with 5% skimmed milk powder for 1 h. Subsequently, PVDF membrane was closed overnight at 4 °C using different primary antibody solutions and washed three times with PBST for 5 min each time; secondary antibody was incubated at room temperature for 1 h and washed five times with PBST. After completion, ECL developer was added, the gel imager (Apeigen, New York, NY, USA) was imaged, and the grayscale values of the bands were analyzed using ImageJ software.

### 4.13. Xenograft Tumor Growth Experiments

Cultured SW620 cells were inoculated subcutaneously into nude mice. Approximately one week later, the mice were randomly divided into 5 groups of 7 mice each, in the following order: control group, 5-FU (25 mg/kg) group, SS (50 mg/kg) group, Ent (25 mg/kg) group and SS (50 mg/kg) + Ent (25 mg/kg) group. Finally, the tumor size and weight were measured and recorded every 7 days. And the tumor size was calculated according to the formula: tumor volume = 0.5 × (length × 2 width).

### 4.14. Immunochemistry

Tumor tissue specimens were isolated and fixed in 4% paraformaldehyde solution for 48 h. Samples were then embedded in paraffin for sectioning, followed by H&E staining and immunohistochemical staining (IHC) by Hengjia Bio (Jilin, China).

### 4.15. Molecular Docking

Crystal structure files of protein targets were downloaded from Uniprot database and PDB database (HDAC1 protein PDB ID: 7SME, HDAC2 protein PDB ID: 6WBZ, HDAC3 protein ID: AF-O15379-F1, HDAC8 protein ID: AF-Q9BY41-F1). The chemical structures of the compounds were obtained from the PubChem database (https://pubchem.ncbi.nlm.nih.gov/, accessed on 1 September 2023); the small molecule structural formulas (PDB format) were drawn for docking using Chem draw 8.0.3 and the compounds were virtually docked to the protein targets using Autodock 4.2.6. The docking results were visualized and analyzed via PyMOL 2.2.0, Discovery Studio Client v19.1.0 software.

### 4.16. Statistical Analysis

All experiments were performed at least three times independently. Data are expressed as mean ± standard deviation. Statistical significance (using graph PadPrism9, * *p* < 0.05, ** *p* < 0.01, *** *p* < 0.001, ^ns^ *p* ≥ 0.05, 0.001).

## 5. Conclusions

**Figure 9:**
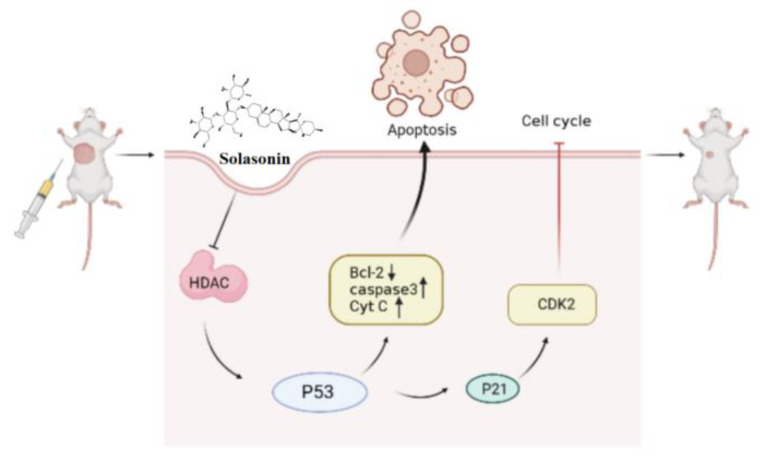
SS activates acetylation of p53 through inhibition of HDAC expression, thereby inducing apoptosis and cycle arrest. Single arrow represents promotion, no arrow represents inhibition.

In summary (Figure 9), the aim of this study was to identify active compounds from *Solanum nigrum* L. that can effectively inhibit CRC growth and to investigate their potential mechanism of action in epigenetic aspects. We found that HDAC is one of the targets of SS. The inhibition of HDAC increased the acetylation of P53, leading to increased apoptosis, accompanied by P21-induced cycle block. This provides new insights into the antitumor effects and mechanisms of SS in CRC.

## Figures and Tables

**Table 1:** Results of docking scores.

	HDAC1	HDAC2	HDAC3	HDAC8
Solasonin	−6.4	−8.09	−7.31	−6.59

## Data Availability

Data will be made available on request.

## Supplementary Materials

The following supporting information can be downloaded at: https://www.mdpi.com/article/10.3390/molecules28186649/s1, Figure S1: The structures of the compounds and ^1^H-NMR and ^13^C-NMR spectra.

## Sample Availability

Available.

## Abbreviations

5-FU: 5-fluorouracil; CCK-8, cell count kit-8; CDK2, cell cycle protein-dependent kinase 2; COAD, colonic adenocarcinoma; CRC, colorectal cancer; Cyt C, Cytochrome C; Ent, Entinostat; HDAC, histone deacetylase; IHC, immunohistochemical staining; NPs, natural products; SS, Solasonin.
