# Supplementary material for: Anti-Colorectal Cancer Activity of Solasonin from Solanum nigrum L. via Histone Deacetylases-Mediated p53 Acetylation Pathway
Source: Molecules. 2023 Sep 15;28(18):6649. doi: 10.3390/molecules28186649 (PMC10534604; doi:10.3390/molecules28186649)
Supplement: Supplementary file 1 [file molecules-28-06649-s001.zip › molecules-2607053-supplementary.pdf]

Firgure S1: The structures of the compounds and 1H-NMR and 13C-NMR spectra.

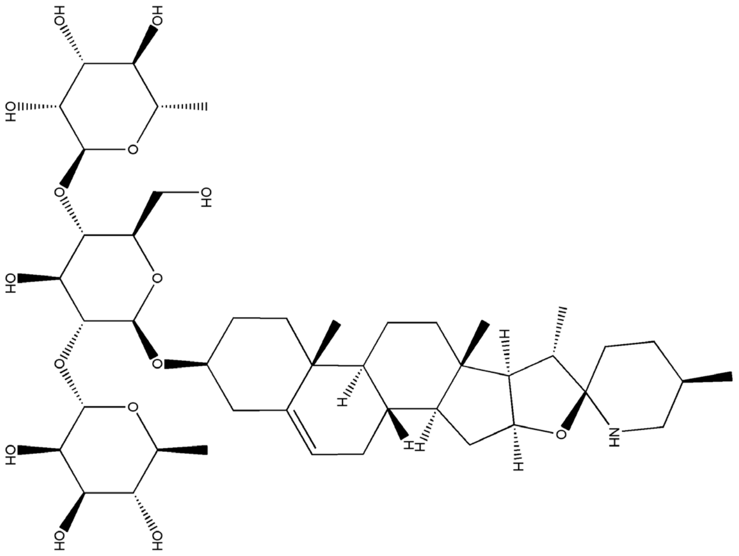

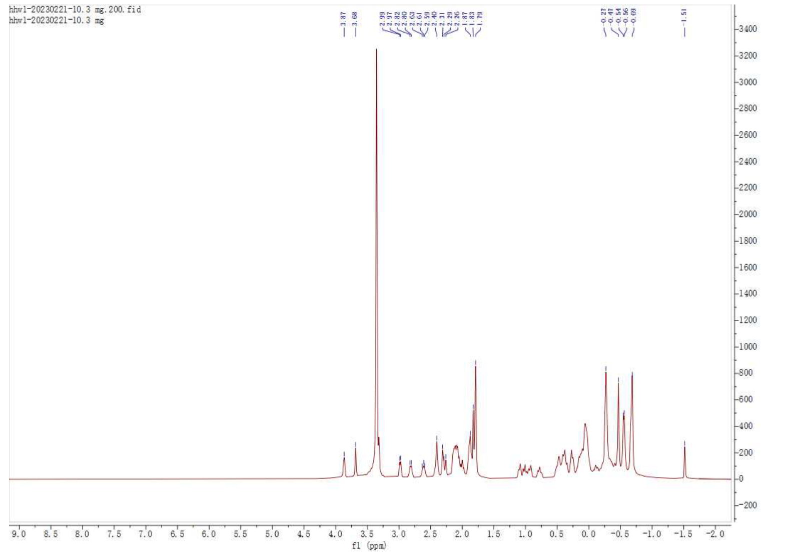

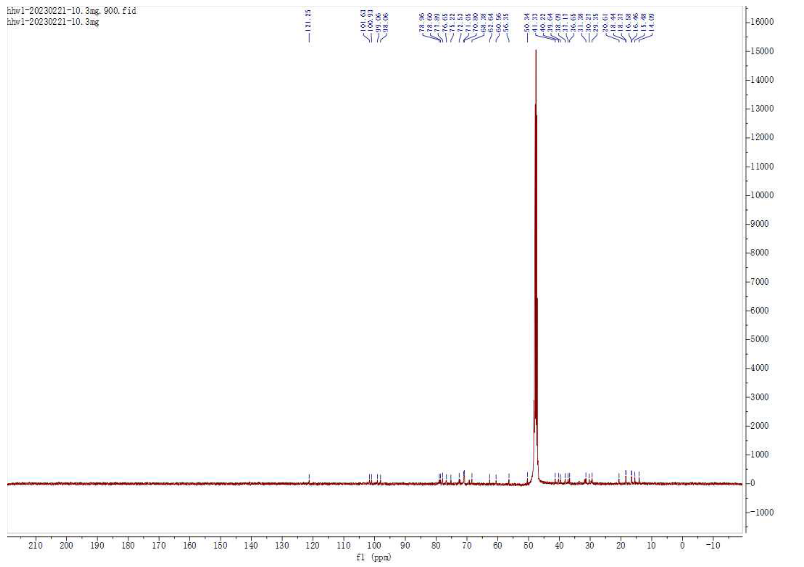

Compound1 ^{1}H-NMR and ^{13}C-NMR

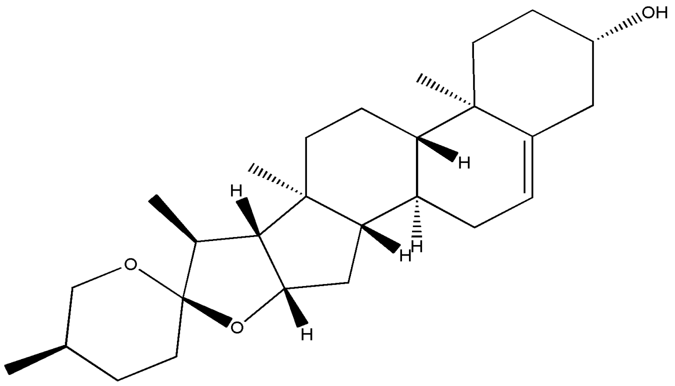

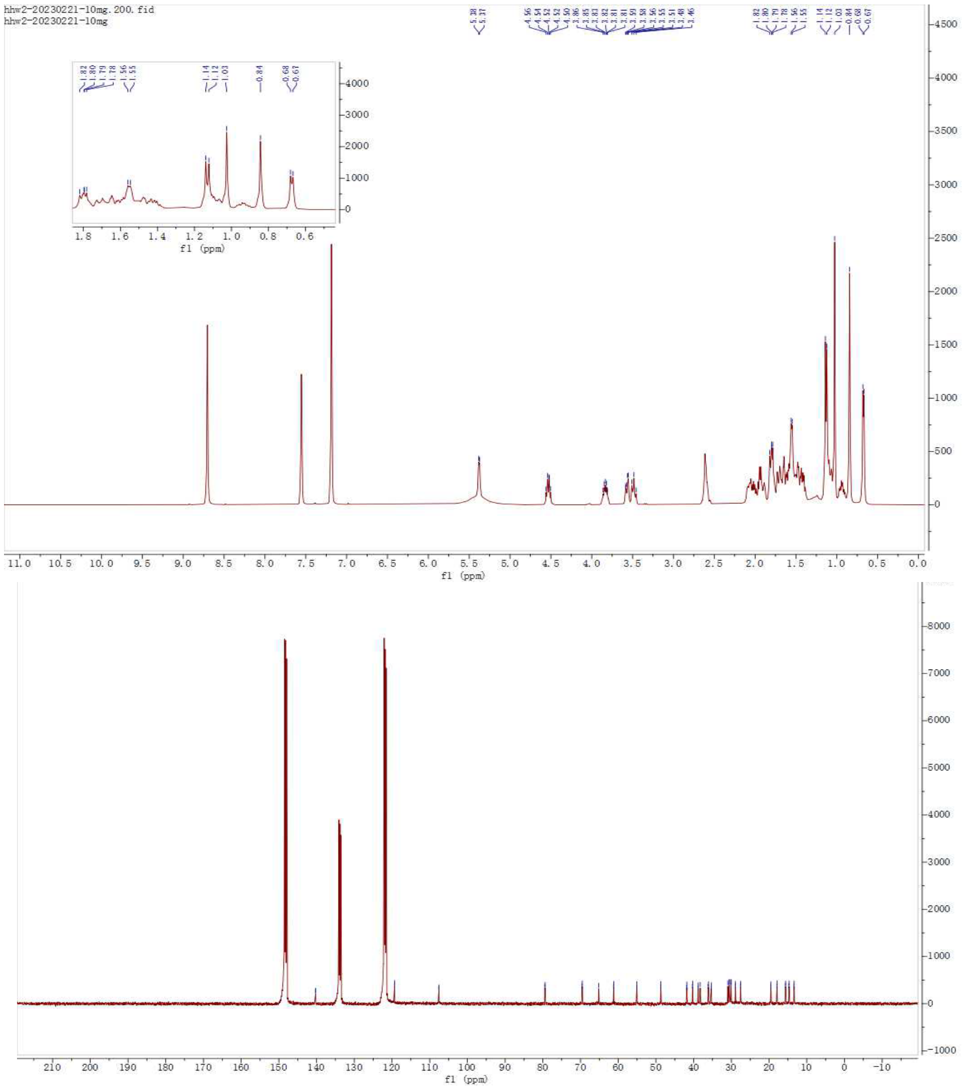

Compound2 ^{1}H-NMR and ^{13}C-NMR

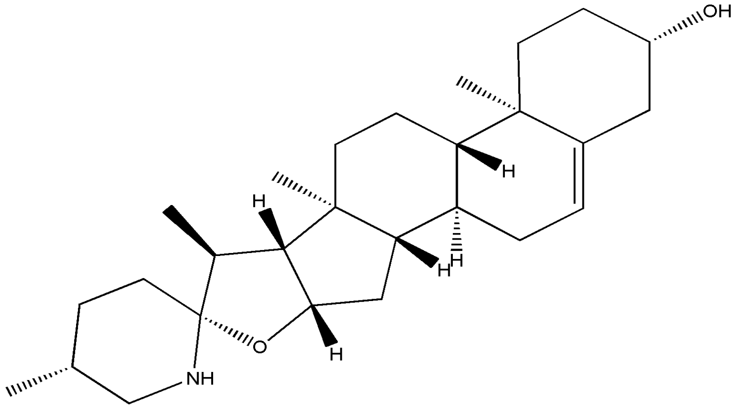

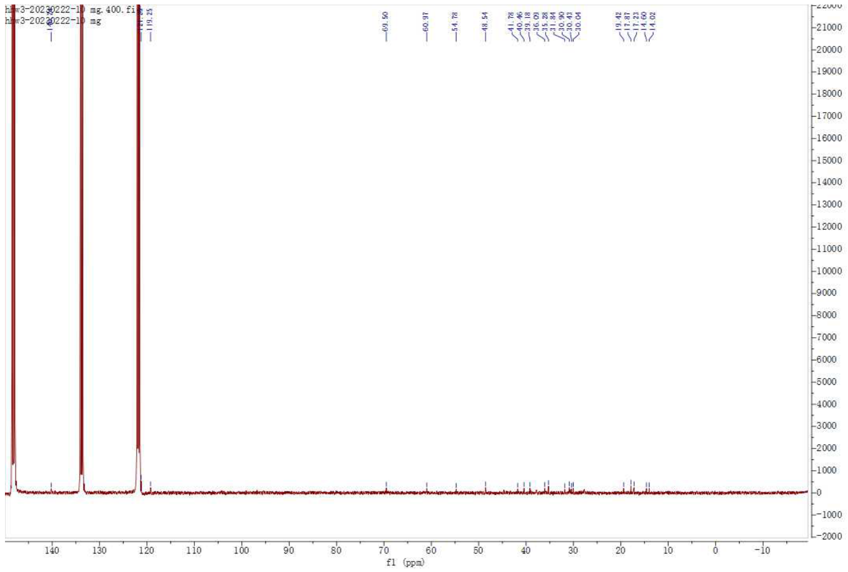

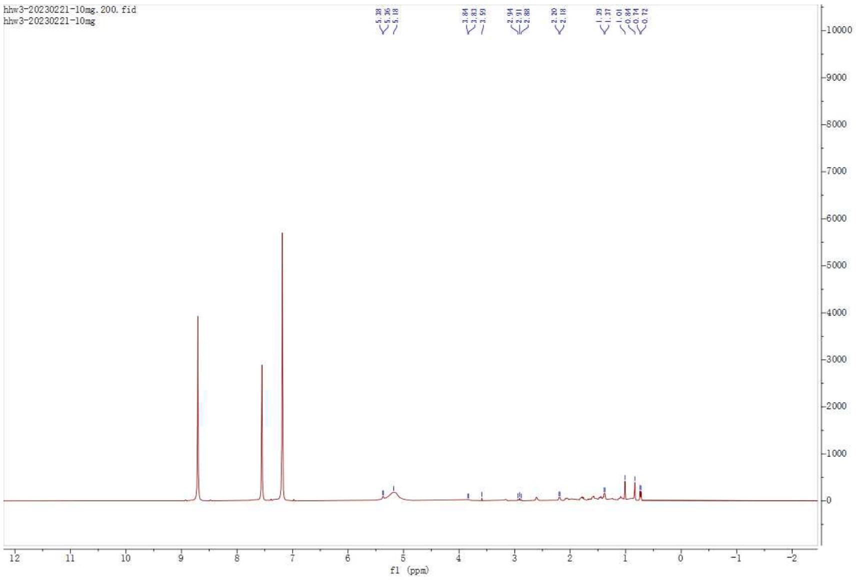

Compound3 ^{1}H-NMR and ^{13}C-NMR

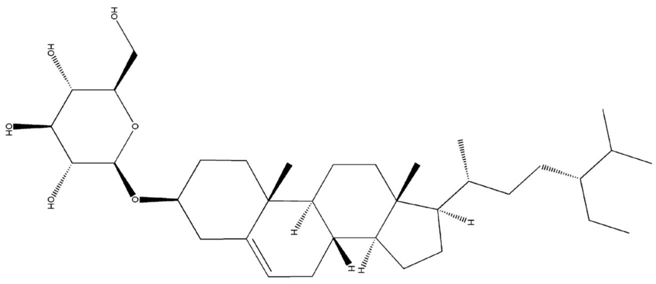

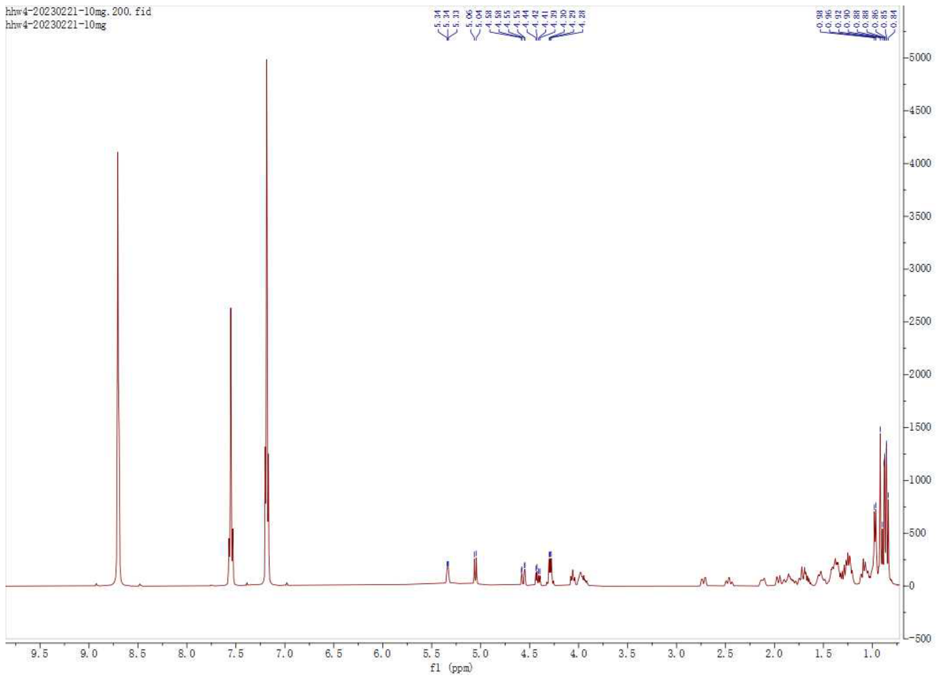

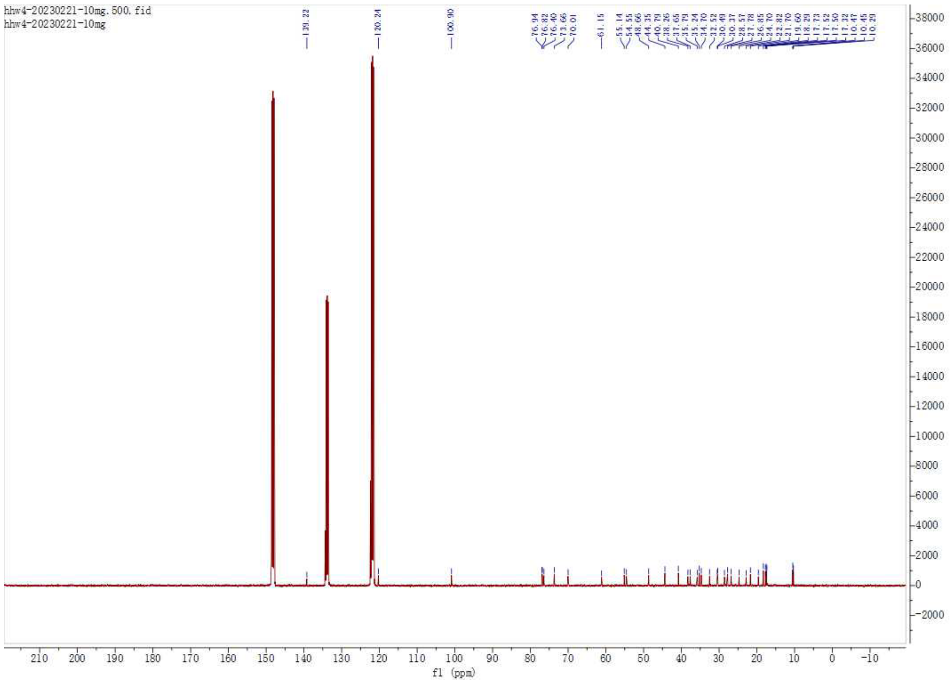

Compound4 ^{1}H-NMR and ^{13}C-NMR

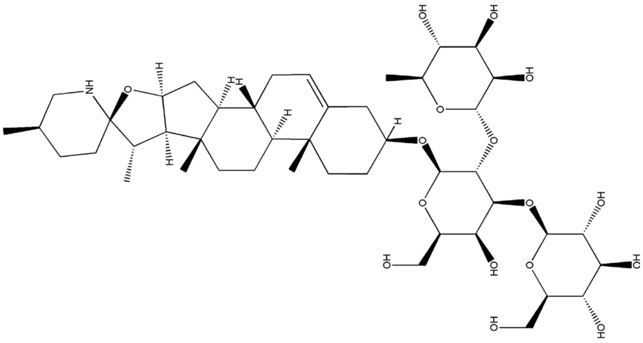

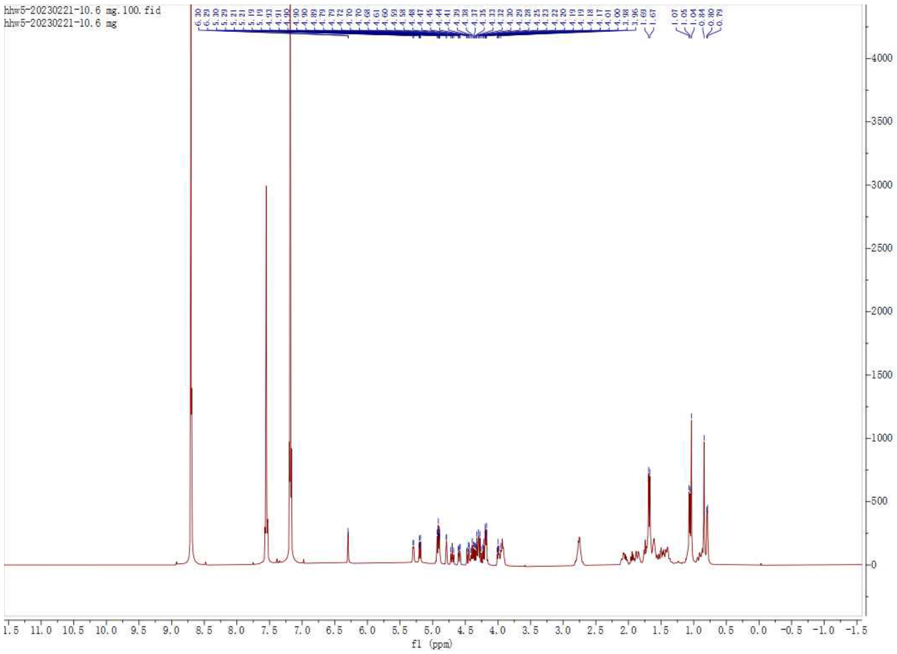

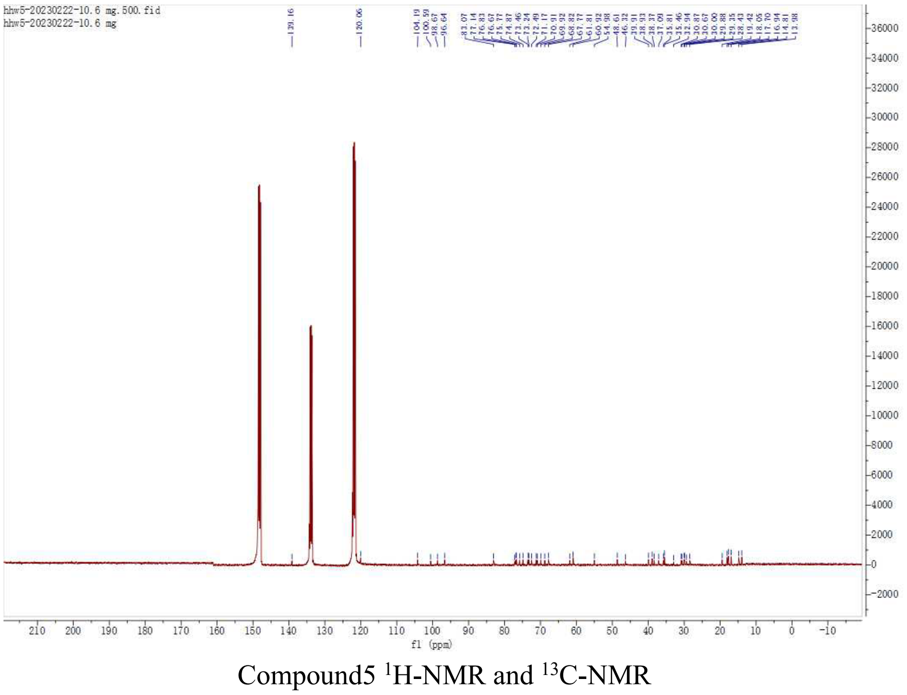

Compound5 ^{1}H-NMR and ^{13}C-NMR

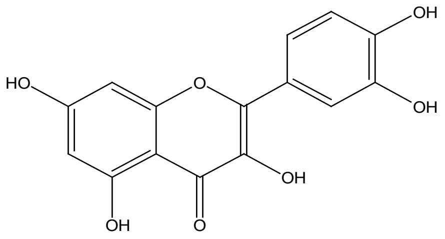

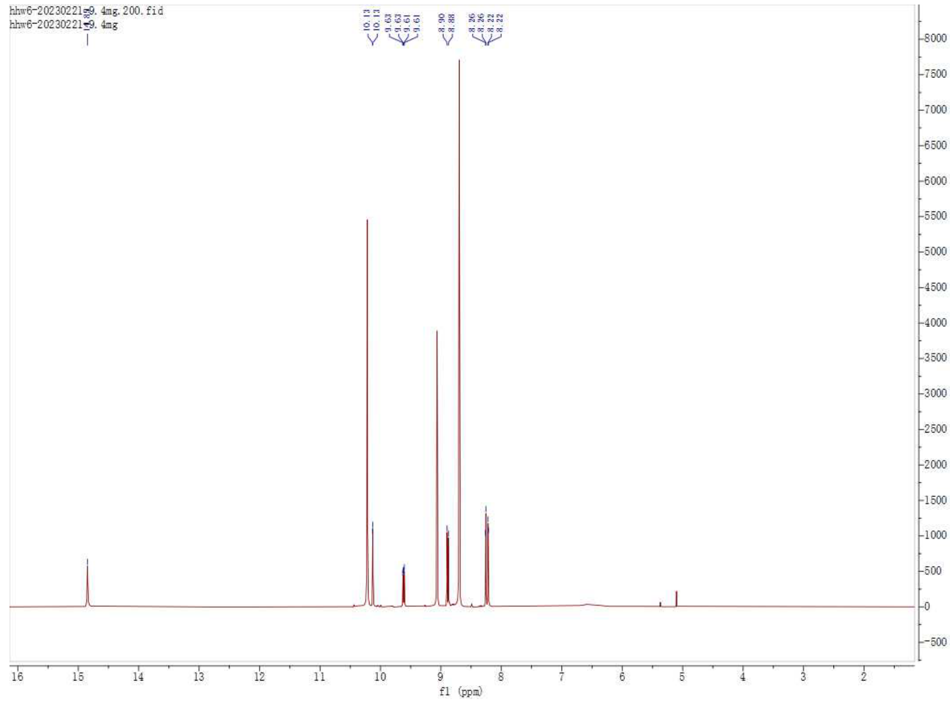

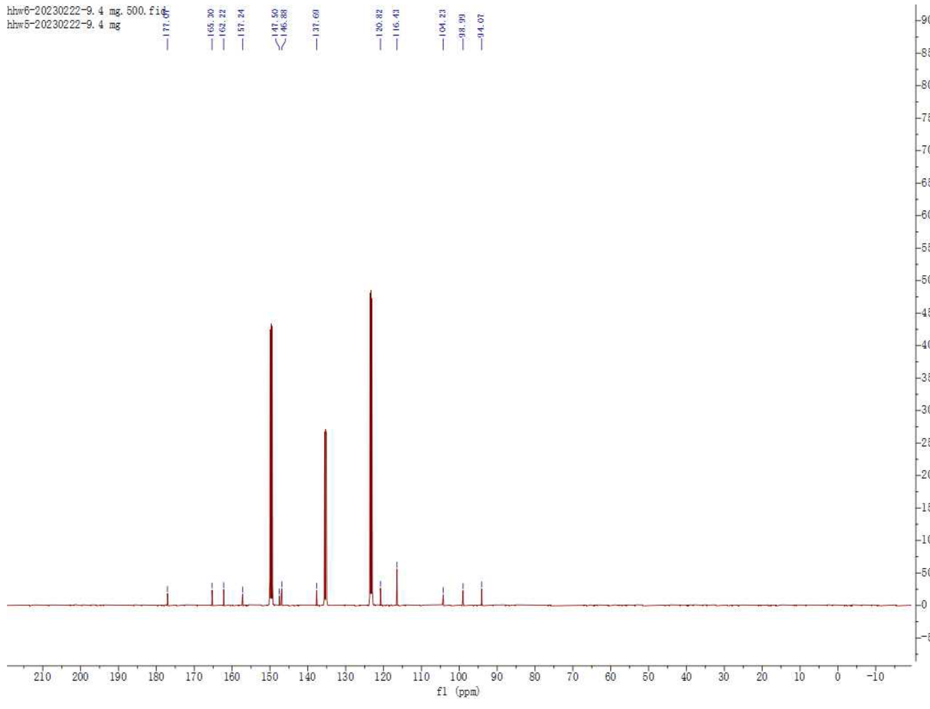

Compound6 ^{1}H-NMR and ^{13}C-NMR

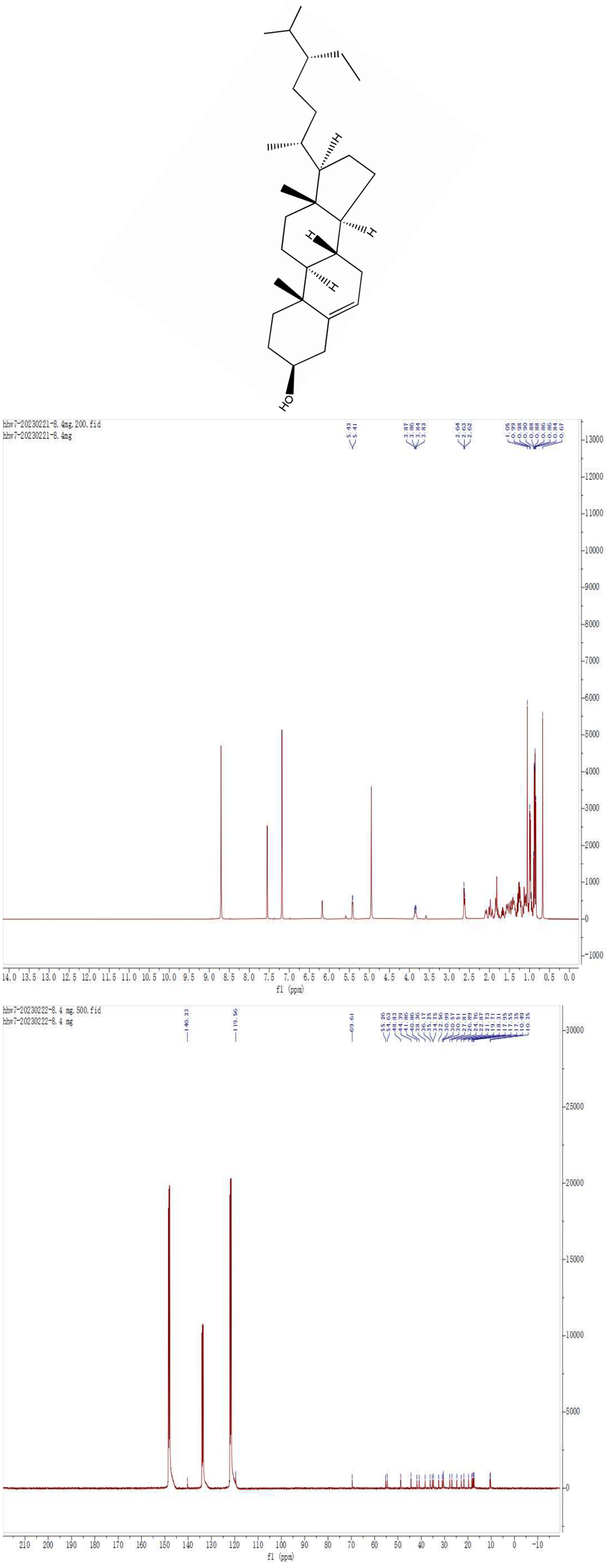

Compound7 ^{1}H-NMR and ^{13}C-NMR
